# Autism risk assessment in siblings of affected children using sex-specific genetic scores

**DOI:** 10.1186/2040-2392-2-17

**Published:** 2011-10-21

**Authors:** Jerome Carayol, Gerard D Schellenberg, Beth Dombroski, Emmanuelle Genin, Francis Rousseau, Geraldine Dawson

**Affiliations:** 1 IntegraGen SA, Evry, France; 2 Department of Pathology and Laboratory Medicine, University of Pennsylvania, Philadelphia, Pennsylvania, USA; 3 INSERM U946, Variabilité Génétique et Maladies Humaines, Fondation Jean Dausset-CEPH, Université Paris Diderot, Paris, France; 4 Autism Speaks and the Department of Psychiatry, University of North Carolina at Chapel Hill, Chapel Hill, North Carolina, USA

**Keywords:** Autism, risk assessment, common variants, genetic score, sex effects

## Abstract

**Background:**

The inheritance pattern in most cases of autism is complex. The risk of autism is increased in siblings of children with autism and previous studies have indicated that the level of risk can be further identified by the accumulation of multiple susceptibility single nucleotide polymorphisms (SNPs) allowing for the identification of a higher-risk subgroup among siblings. As a result of the sex difference in the prevalence of autism, we explored the potential for identifying sex-specific autism susceptibility SNPs in siblings of children with autism and the ability to develop a sex-specific risk assessment genetic scoring system.

**Methods:**

SNPs were chosen from genes known to be associated with autism. These markers were evaluated using an exploratory sample of 480 families from the Autism Genetic Resource Exchange (AGRE) repository. A reproducibility index (RI) was proposed and calculated in all children with autism and in males and females separately. Differing genetic scoring models were then constructed to develop a sex-specific genetic score model designed to identify individuals with a higher risk of autism. The ability of the genetic scores to identify high-risk children was then evaluated and replicated in an independent sample of 351 affected and 90 unaffected siblings from families with at least 1 child with autism.

**Results:**

We identified three risk SNPs that had a high RI in males, two SNPs with a high RI in females, and three SNPs with a high RI in both sexes. Using these results, genetic scoring models for males and females were developed which demonstrated a significant association with autism (*P *= 2.2 × 10^-6 ^and 1.9 × 10^-5^, respectively).

**Conclusions:**

Our results demonstrate that individual susceptibility associated SNPs for autism may have important differential sex effects. We also show that a sex-specific risk score based on the presence of multiple susceptibility associated SNPs allow for the identification of subgroups of siblings of children with autism who have a significantly higher risk of autism.

## Background

Autistic disorder is the most severe form of a group of autism spectrum disorders (ASDs) characterized by impairments in social interaction, deficits in verbal and non-verbal communication, restricted interests, and repetitive behaviors [1]. With a prevalence of 1 in 110 children, ASDs are among the most common forms of severe developmental disability [2]. The average recurrence risk of autism in siblings of affected children is approximately 10% [3]. This rate is much higher than the prevalence rate for ASDs in the general population, but lower than would be expected for a highly penetrant mutation in a mendelian disorder [4].

The inheritance pattern of autism in most families is complex and not compatible with simple Mendelian inheritance [5,6]. There is significant interest in the early identification of infants at higher risk for autism because studies have shown that early intervention leads to significantly improved long-term outcome for the whole family [7,8]. Several common variants localized in biological and positional (that is, under known linkage peaks) candidate genes have been associated with autism and some have been replicated in independent studies [9]. Further support for these associations comes from genes for which, in addition to autism-associated common variants, rare mutations and/or copy number variations (CNVs) have been shown to contribute to the disease, and/or for which gene-disrupted mice exhibited autism-like traits. These genes include *CNTNAP2 *[10-13], *RELN *[14-19] and *GABRB3 *[20-23].

When taken individually, the risk of autism associated with variants remains modest, but Carayol *et al*. [24] recently showed that the accumulation of multiple risk alleles markedly increases the risk of autism in siblings of children who have been diagnosed with autism. They proposed a genetic score (GS) that, compared with studying polymorphisms individually, improves the identification of subgroups of individuals at greater risk of autism [24]. In the case of autism, tools for genetic risk assessment are highly desirable to complement available behavioral assessments.

Another important characteristic of autism is the sex difference with a 4.5:1 male to female ratio [2]. Second, intellectual disability, a key clinical dimension associated with outcome, is more frequent in females than males [25]. Third, the risk of epilepsy is 18 times higher in females than males [26]. This sex difference may partly be explained by sex-specific risk alleles or genes with different expression or activity based on sex [27,28].

In the present study we propose to improve the genetic risk score model developed by Carayol *et al*. [24] by adding additional SNPs filtered for their relative importance using internal validation process and by also developing separate sex-specific genetic risk scores for males and females using a first sample of families with children with autism (exploratory sample). Their ability to better identify siblings of children with autism who are at high risk of autism was then evaluated and replicated in an independent second sample of autism families (replication sample).

## Methods

The study design involved two independent family samples. The first sample (the 'exploratory' sample) consisted of 480 families from the Autism Genetic Resource Exchange (AGRE; http://www.agre.org) repository with at least 1 sibling diagnosed with a 'strict' definition of autism according to the Autism Diagnostic Interview Revisited (ADI-R) and no unaffected siblings. A total of 844 affected siblings including 664 males and 179 females met the diagnostic criteria for 'strict' autism. Minimizing phenotypic heterogeneity can lead to an improvement of the study power [29]. Shao *et al*. [30] demonstrated that the use of homogeneous phenotype increases the power of linkage studies in autism. Linkage signals have been observed in studies in which the samples were stratified according to specific phenotypes such as the sex [28,31,32], delayed onset of phrase speech [30,33,34], and severe obsessive-compulsive behaviors [35]. Two genome-wide association studies using overlapping samples of children with autism identified two different common variants in *CNTNAP2*, a gene localized in the 7q34-7q36 region linked to language disability in autism [36]; one SNP has been associated with autism through the use of the quantitative trait 'age at first word' [10] and the other using a qualitative strict autism diagnosis [11]. Similarly, a recent genome-wide association study (GWAS) [37] reported the largest association with autism in *MACROD2 *using the strict autism diagnosis. Therefore, as in Shao *et al*. [30], we studied individuals with a strict autism rather than the heterogeneous broad autism spectrum disorder phenotype. The second sample (the 'replication' sample) included 187 families consisting of the 2 parents, at least 1 child with autism and 1 unaffected sibling from a sample collection at the University of Pennsylvania. This replication sample led to 351 children with autism (291 males and 60 females) with the same strict definition of the disease and 90 unaffected children (39 males and 51 females). Ethnicity was self-reported by parents as Caucasian, Asian, Hispanic or Latino, Black or African American, Native Hawaiian or other Pacific Islander, or of mixed ethnicity. Caucasians represented the major ethnicity, with more than two-thirds of families in each sample.

Ten autism susceptibility genes were selected for this study. Four of them (*PITX1*, *EN2*, *SLC25A12 *and *ATP2B2*) have been previously demonstrated to have a predictive ability and were used in a genetic score-based model [24]. Genes shown to be statistically associated with autism in at least one study using AGRE collection, even at the nominal level, and for which additional data support their implication in autism, were also included. Six genes fulfilled the statistical association condition, four of which were replicated in one or more independent study: *HOXA1 *[38,39], *GRIK2 *[40-42], *ITGB3 *[43-46] and *CNTNAP2 *[10,11]; one gene, *MARK1*, was found to be significantly overexpressed in brain from individuals with autism compared to unaffected individuals [47] and the last gene, *JARID2 *was chosen since one SNP, rs7766973, displays the strongest association with autism (*P *= 6.8 × 10^-7 ^[48]) among the three GWAS performed on AGRE family data [37,42,48]. Table 1 lists the genes selected for the study and the associated SNPs with their deleterious alleles and corresponding frequencies.

**Table 1:** Risk allele frequency (defined as the allele associated with autism)

Gene	SNP	Risk allele	Exploratory sample		Replication sample	
			
			Frequency	HWE^a^	Frequency	HWE^a^
*MARK1*	rs12410279	A	0.85	0.26	0.83	1.00

*SLC25A12*	rs2292813	C	0.90	1.00	NE^b^	NE

*ATP2B2*	rs2278556	A	0.40	0.68	0.38	0.11

*PITX1*	rs6872664	C	0.89	1.00	0.85	0.32

*GRIK2*	rs2235076	G	0.98	1.00	NE	NE

*HOXA1*	rs10951154	T	0.86	0.02	0.86	1.00

*CNTNAP2*	rs7794745	T	0.40	0.73	0.39	0.04

*EN2*	rs1861972	A	0.73	0.68	0.76	0.90

*ITGB3*	rs5918	T	0.87	1.00	0.85	0.85

*JARID2*	rs7766973	C	0.60	0.22	0.58	0.76

^a^Hardy-Weinberg Equilibrium (HWE) *P *value estimated with the exact test [65].

^b^NE, not estimated since not genotyped in the replication sample.

SNP, single nucleotide polymorphism.

All parents and children from the exploratory sample were genotyped for these ten markers. Only SNPs that were selected for further investigation were genotyped in the replication sample. Genotyping was performed using TaqMan allele discrimination assays (Applied Biosystems, Foster City, CA, USA). Genotyping was performed in 384-well plates with 5 ng genomic DNA, 0.075 μl of 20 × SNP TaqMan Assay mix, 1.5 μl of TaqMan Universal PCR Master Mix and 1.425 μl of dH_2_O in each well. PCR was performed at 95°C for 10 min, followed by 50 cycles at 92°C for 15 s and 60°C for 90 s (9700 Gene Amp PCR System; Applied Biosystems). Plates were then subjected to endpoint reading (7900 Real-Time PCR System; Applied Biosystems). The alleles were called automatically using the SDS software (Applied Biosystems), and a visual inspection of genotype clusters was performed. Genotyping quality was assessed by signal intensity plots and missing genotype frequencies; any sample with poor clustering and missing fractions ≥5% per SNP were retyped. Parental genotypes were used to investigate Hardy-Weinberg equilibrium (HWE) and to check for Mendelian inconsistencies. Families with remaining inconsistencies were excluded.

The development of the genetic score model and the definition of the increased risk GS thresholds (that define the high-risk groups) were based on the exploratory sample with all affected children whereas, for the replication study using the second sample, the index cases were excluded.

A model that is efficient only in the sample in which it was developed does not have validity. To be valid, the results need to be reproduced in a separate independent population. A genetic score model, such as the one proposed in this paper, is generally built on the simple sum of deleterious alleles observed at each of the chosen genes. Thus, the reproducibility of the genetic score is conditioned by the reproducibility of the deleterious allele for each SNPs included in the model. Markers that are more reproducible carry stronger and more stable information. The reproducibility of the SNPs was analyzed using the bootstrap resampling process and a reproducibility index (RI) was estimated similarly to Ma [49] as follows: (1) generation of a 'pseudosample' consisting of 480 families by randomly sampling the 480 families of the exploratory population with replacement; (2) estimation of the genetic relative risk associated with the deleterious allele of each SNP as defined in Table 1; (3) repetition 1,000 times of steps 1 and 2; (4) estimation for each SNP of the RIs indicating the proportion of 'pseudosamples' in which the deleterious allele maintains a risk greater than 1.00 in males, in females or in both males and females.

A high RI indicates that the effect of a deleterious allele of a given SNP is maintained across the bootstrap pseudosamples and that this SNP is a good candidate for the reproducibility of the genetic score. A stringent RI = 0.80 in children with autism was set to select best SNPs. Then, the RI in males and females with autism was checked separately to discard SNPs that lack of stability in a particular sex. Since all variants have been associated with autism using AGRE family data, this internal validation process prevents from an optimistic evaluation of their association, that is, an overestimation of the effect of risk alleles, and a potential deterioration of this effect in an independent sample. The sex genetic scores (GS) was then constructed as follows:

$$\text{G}{\text{S}}_{\text{sex}}={\text{W}}_{\text{all}}\cdot \text{R}{\text{S}}_{\text{all}}+{\text{W}}_{\text{sex}}\cdot \;\text{R}{\text{S}}_{\text{sex}}$$

where sex = (male, female); RS_all _and RS_sex _are the risk scores built as the sum of deleterious alleles from genes with a high RI in males only (RS_male_), in females only (RS_female_) or in both sexes (RS_all_); and W_all_, W_male_, and W_female _are the integer values of the corresponding genetic relative risks (GRR) associated with the corresponding risk scores (RS_all_, RS_male _and RS_female_, respectively). These weights were calculated following Lin *et al*. [50] who showed that a weighted genetic score provided more predictive value than an unweighted genetic score.

Because the exploratory sample did not include unaffected children, all genetic relative risks were estimated as described in Carayol *et al*. [24] using the case-pseudocontrol approach proposed by Cordell and Clayton [51] and implemented in the DGCgenetics R package (http://www-gene.cimr.cam.ac.uk/clayton/software/). Sensitivity and specificity values of the GSs were estimated in the exploratory and the replication samples as in Carayol *et al*. [24]. Areas under the receiver operating curves (AUCs) were estimated in the exploratory sample and tested against the AUC = 0.5 null hypothesis to validate the discriminative power of the GSs. However, AUCs do not provide an informative tool of the clinical utility of the genetic score (here, the high-risk classification of siblings of children with autism). Cutoff values were chosen to define a high-risk group in the exploratory sample and the odds ratios were estimated. These high-risk thresholds (one for male and one for female) were selected considering a false positive rate lower than 20% (that is, specificity higher than 80%). External validation of the clinical utility of the high-risk GS group was then conducted in the replication sample. Positive predictive values in siblings of children with autism were estimated from the sensitivity, specificity and the sibling recurrence risk estimates in males and females. Since no data were available in the literature, we estimated the sibling recurrence risk to 0.16 in males and 0.04 in females assuming an overall 0.10 sibling recurrence risk [3] and a 4:1 male to female sex ratio [2].

## Results

None of the SNPs exhibited a departure from HWE and allele frequencies were similar between samples (Table 1). Table 2 lists the RI of each SNP based on the bootstrap analysis using the exploratory sample. Eight markers reached the stringent 80% RI threshold. SNPs rs2292813 (*SLC25A12*) and rs2235076 (*GRIK2*) were excluded because of their low reproducibility (RI = 52% and 36%, respectively). Among the eight remaining SNPs, two displayed low RI in males but RI of 100% in females, rs12410279 (*MARK1*, RI_male _= 47%) and rs5918 (*ITGB3*, RI_male _= 65%). Inversely, three SNPs displayed a low RI in females and RI greater than 95% in males, rs227855 (*ATP2B2*, RI_female _= 59%), rs6872664 (*PITX1*, RI_female _= 30%) and rs10951154 (*HOXA1*, RI_female _= 20%).

**Table 2:** Reproducibility indexes (RIs) in children with autism, in males and in females

Gene	SNP	RI in children with autism	RI in male children with autism	RI in female children with autism
*MARK1*	rs12410279	**0.93**	0.468	**1.00**

*SLC25A12*	rs2292813	0.52	0.757	0.52

*ATP2B2*	rs2278556	**0.99**	**0.997**	0.59

*PITX1*	rs6872664	**0.97**	**0.983**	0.30

*GRIK2*	rs2235076	0.36	0.277	0.59

*HOXA1*	rs10951154	**0.93**	**0.958**	0.20

*CNTNAP2*	rs7794745	**1.000**	**1.000**	**0.89**

*EN2*	rs1861972	**0.97**	**0.880**	**0.94**

*ITGB3*	rs5918	**0.98**	0.646	**1.00**

*JARID2*	rs7766973	**0.98**	**0.951**	**0.88**

RIs that reached the 80% threshold are in bold.

SNP, single nucleotide polymorphism.

The three separate risk scores were then constructed based on the sum of deleterious alleles in their corresponding SNPs. These included rs7794745, rs1861972 and rs7766973 for RS_all_, rs12410279 and rs5918 for RS_female_, and rs2278556, rs6872664 and rs10951154 for RS_male_. The GRRs associated to one point increase in the RS were estimated to be 1.23 for RS_all _(*P *= 2.3 × 10^-5^; 95% confidence interval (CI) 1.12 to 1.36), 1.25 for RS_male _(*P *= 5.8 × 10^-4^; 95% CI 1.10 to 1.41) and 2.29 for RS_female _(*P *= 1.7 × 10^-6^; 95% CI 1.57 to 3.34). The overall *P *value of the three tested scores were 3.1 × 10^-9 ^with corresponding weights of 1.00, 1.00 and 2.00 for RS_all_, RS_male _and RS_female_, respectively. The two genetic scores (GSs) were then constructed. GS_male _ranged between 3 and 12 with a GRR associated to 1 point increase in the score of 1.23 (*P *= 2.2 × 10^-6^; 95% CI 1.13 to 1.34) and GS_female _ranged between 4 and 14 with a GRR of 1.41 (*P *= 1.9 × 10^-5^; 95% CI 1.21 to 1.65) for a highly significant global test with *P *= 8.4 × 10^-10^. Table 3 displays the sensitivity and specificity values for the GS in males and females. To define the high-risk group, GS values were selected in males and females with the aim to minimize the number of false positive below 20% and to maximize the sensitivity as high as possible. A genetic score threshold of nine points for males was associated with a moderate 0.24 sensitivity (95% CI 0.19 to 0.28) and a 0.86 specificity (95% CI 0.82 to 0.90) that minimizes the number of false positive test to 0.14 and lead to a 0.23 positive predictive value (PPV). For females, a genetic score threshold of 12 was associated with a similar specificity of 0.86 (95% CI 0.80 to 0.92) but a higher sensitivity of 0.37 (95% CI 0.29 to 0.44) and a PPV of 0.09. These two GS values were chosen as thresholds to define the group of children with a high risk of autism. AUCs were estimated to be 0.59 and 0.66 in males and females, respectively. They are both significantly different from the 0.5 null hypothesis (*P *= 2 × 10^-8 ^and 1.5 × 10^-7^) indicating a predictive ability of the GSs.

**Table 3:** Genetic score (GS) sensitivities and specificities with their 95% CIs by sex estimated in the exploratory sample

Genetic score threshold	Males		Females	
	
	Sensitivity (95% CI)	Specificity (95% CI)	Sensitivity (95% CI)	Specificity (95% CI)
3	1.00	0.000	-	-

4	1.00 (0.99 to 1.00)	0.01 (0.01 to 0.02)	1.00	0.00

5	0.97 (0.94 to 1.00)	0.03 (0.02 to 0.05)	1.00	0.00

6	0.90 (0.85 to 0.94)	0.19 (0.15 to 0.22)	1.00	0.00

7	0.75 (0.70 to 0.80)	0.41 (0.36 to 0.46)	1.00	0.00

8	0.47 (0.43 to 0.52)	0.64 (0.59 to 0.69)	0.99 (0.98 to 1.00)	0.10 (0.00 to 0.19)

9	**0.24 (0.19 to 0.28)**	**0.86 (0.82 to 0.90)**	0.90 (0.85 to 0.96)	0.20 (0.15 to 0.25)

10	0.08 (0.06 to 0.11)	0.95 (0.92 to 0.97)	0.78 (0.71 to 0.85)	0.40 (0.31 to 0.49)

11	0.02 (0.01 to 0.04)	0.98 (0.96 to 0.99)	0.61 (0.52 to 0.69)	0.65 (0.57 to 0.74)

12	0.00	1.00	**0.37 (0.29 to 0.44)**	**0.86 (0.80 to 0.92)**

13	-	-	0.17 (0.11 to 0.23)	0.94 (0.89 to 0.98)

14	-	-	0.03 (0.01 to 0.06)	0.99 (0.97 to 1.00)

The two GSs chosen as threshold value to define children with a higher risk of autism in males and in females are shown in bold.

In the replication sample (Table 4), sensitivity and specificity associated with the high-risk group GS threshold (GS_male _= 9) were slightly higher in males (but not significantly different as it can be seen from the overlapping 95% CIs) with a 0.26 (95% CI 0.18 to 0.35) sensitivity and 0.87 (95% CI 0.76 to 0.98) specificity. The PPV reached 0.28 for a 0.16 sibling recurrence risk. Differences were observed in females for the sensitivity with an estimated 0.28 (95% CI 0.12 to 0.44) instead of 0.37 and the specificity with a 0.76 specificity (95% CI 0.64 to 0.89) instead of 0.86 but the differences were not significant (overlapping confidence intervals). In females, variances for sensitivity and specificity values were larger in the replication sample than in the exploratory sample because of the smaller number of females in the replication sample. As a consequence, the PPV (estimated to 5%) was very small and close to the 4% sibling recurrence risk.

**Table 4:** Sensitivity and specificity estimates in the exploratory and replication samples with their corresponding 95% CIs for the high-risk group

	Exploratory sample	Replication sample
Males:		

Sensitivity	0.24 (0.19 to 0.28)	0.26 (0.18 to 0.35)

Specificity	0.86 (0.82 to 0.90)	0.87 (0.76 to 0.98)

Females:		

Sensitivity	0.37 (0.29 to 0.44)	0.28 (0.12 to 0.44)

Specificity	0.86 (0.80 to 0.92)	0.76 (0.64 to 0.89)

Extending the analysis to a broader definition of autism and including or excluding the index cases as was performed with the replication study did not change the characteristics of the genetic score or the associated significance levels.

## Discussion

Our results demonstrate that the sex difference in autism may have an important influence on the genetic score characteristics, and therefore, on the risk assessment. Taking sex and reproducibility of the SNPs into account led to two GSs with different characteristics that allowed the identification of a subgroup of siblings of children with autism with a high risk of autism in males. The genetic score model with four genes [24] was also tested on this large sample of families and its association was clearly lower (*P *= 7 × 10^-4 ^in males and females as a whole) compared to those of the sex-specific GSs (*P *= 2.2 × 10^-6 ^and 1.9 × 10^-5 ^for males and females, respectively). The risk for males with a high GS to develop autism was 28%, almost three times higher than the reported 10% sibling recurrence risk. In females, the 10% recurrence risk seems overestimated and we estimate this value to 4% considering a 4.5:1 male to female sex ratio.

The GS model has been developed through the use of affected children and the pseudocontrol approach [52,53]. This was confirmed by analyzing unaffected siblings of children with autism. The pseudocontrols approach has been validated for the estimation of diagnostic accuracy using only affected children compared to full population-based data [54]. We cannot exclude an over-representation of deleterious alleles in unaffected siblings compared to pseudocontrols, which are genetically the opposite of affected children, nor the effect of population controls that may lower the risk ratio between affected and unaffected siblings and consequently affect the discriminative ability of the GS models. This does not seem to occur for males since the high-risk class replicates its predictive accuracy but would need further investigation for females.

Reproducibility of effects is of major interest to enter in a predictive model since it conditions the reproducibility of the predictive model outside the study sample, which is of primary importance to validate such a model. According to the replication of the performance of the risk assessment model in males in an independent sample and the ability to find support for female specific variants despite the relatively small number of samples, the proposed approach can be used for developing stable and reproducible models. *SLC25A12 *associated and replicated in different studies [55-58] did not reach the reproducibility thresholds, whereas *JARID2 *that reached a suggestive significant threshold in a unique GWAS [48] seems of more interest. Some markers were reproducible (high RI) in a specific sex only but did not show any statistically significant interaction with sex nor were reported as being sex specific in the literature. The SNP rs7794745 located within *CNTNAP2 *has a high RI in both sexes whereas a previous association with autism has been reported preferentially in males [10,11]. Due to the low number of females analyzed, these studies lack power to observe any association in females [11]. Another SNP, rs5918 located within *ITGB3*, has been shown to be associated with autism in both sexes but with different risk effect [46], which could explain the difference of reproducibility observed in males and females. The stability is not necessarily linked to the sex specificity of the SNP or to the strength of previous association results. This may be explained in part by a study of Jakobsdotir *et al*. [59] which showed that a highly significant association of genes with a disease does not guarantee an effective discrimination between cases and controls.

Several limits of the study may be identified. The moderate number of females with autism in the replication sample as a consequence of the significant sex ratio in autism led to a lack of power for the replication of the high-risk group characteristics. Sibling recurrence risk of males and females were not estimated or reported from real data but calculated assuming a sibling recurrence risk of 10% [3] and the widely observed 4.5:1 male to female sex ratio. Reported PPVs are intuitive estimates that quantify the increase in the risk for an individual (a sibling of a child with autism) who has a genetic score that falls in the high-risk class. Accurate PPVs could be estimated by using observed and reported data. The selection of the genes and the SNPs included in the genetic scores could be discussed. The methodology used to select the common variants and the internal validation approach performed in this study strongly support the implication of these SNPs in autism as well as their discriminative ability. The addition of other SNPs from the same genetic region would have led to a much more complicated model because of the linkage disequilibrium (LD) between these SNPs as well as the haplotypes resulting from the different combination of alleles. Finally, other approaches may be used to select genes to enter in a genetic score. Genes may be selected using statistically significant results from GWAS [60,61] or a complementary approach as in convergent functional genomics (CFG) autism [62,63], when none or few association results reach significance as it is frequently the case in complex disease and particularly in autism.

The recent paper of Lu and Cantor [64] together with the present results highlights the importance of the sex in genetic study of autism. They showed that using sex as a risk factor in GWAS of multiplex autism families increased the power of the study and identified one new gene implicated in calcium channel defect. Stone *et al*. [28] also suggested that sex is an important factor in the genetics of autism and could be used to decrease heterogeneity in genetic study.

## Conclusions

The results of this study confirm previous results [24] that predictive models are of major interest in autism and may help to identify siblings of children with autism at high risk of disease. The choice of genes to enter in the model must be made with caution since association and replication of a particular SNP in different studies are not sufficient justification to enter a SNP in a genetic score and sex is an important factor that needs to be included in autism risk evaluation.

## Competing interests

JC and FR are currently salaried employees of IntegraGen SA and have stock options and patent applications with IntegraGen. GDS, BD and GD declare that they have no competing interests. EG is a consultant for IntegraGen SA.

## Authors' contributions

JC, FR and GDS conceived and designed the experiments. FR, BD and GDS performed the experiments. JC analyzed the data and draft the manuscript. EG validated the statistical method. JC, FR and GDS contributed reagents, materials and/or analysis tools. GD and GS contributed to the collection of the University of Pennsylvanian sample. All coauthors assisted with writing of the manuscript. All authors read and approved the final manuscript.
